# The Attractiveness of B7-H3 as a Target for Lung Cancer Treatment

**DOI:** 10.3390/cancers17091546

**Published:** 2025-05-01

**Authors:** Kostas A. Papavassiliou, Amalia A. Sofianidi, Athanasios G. Papavassiliou

**Affiliations:** 1 First University Department of Respiratory Medicine, ‘Sotiria’ Chest Hospital, Medical School, National and Kapodistrian University of Athens, 11527 Athens, Greece; konpapav@med.uoa.gr; 2 Department of Biological Chemistry, Medical School, National and Kapodistrian University of Athens, 11527 Athens, Greece; amaliasof@med.uoa.gr

In 2015, the U.S. Food and Drug Administration (FDA) approved nivolumab, a programmed cell death protein-1 (PD-1) immune checkpoint inhibitor (ICI), for squamous non-small-cell lung cancer (NSCLC) treatment, making it the first immunotherapy officially approved for lung cancer [1]. During the past 10 years, immunotherapy has revolutionized the therapeutic landscape of lung cancer. However, the overall response rate of current immunotherapy options in NSCLC is only 20–30% [2,3], while acquired resistance to current ICIs is ubiquitous, occurring in more than 60% of initial responders [4]. Novel immune checkpoint molecules are urgently needed in order to strengthen our armamentarium against this deadly disease.

B7 homolog 3 protein (B7-H3), also known as cluster of differentiation 276 (CD276), is a transmembrane protein, a member of the B7 family of immune checkpoint proteins, and closely related to programmed death receptor ligand 1 (PD-L1, also termed B7-H1) [5]. B7-H3 expression in healthy tissues is limited to immune cells [5]; nevertheless, B7-H3 is aberrantly expressed on tumor cells, tumor vasculature, and other tumor microenvironment (TME) components [5]. Notably, B7-H3 expression was detected in cancer-associated fibroblasts (CAFs) in renal cell carcinoma tissue [6].

Research has recently established the expression of B7-H3 across various human malignancies, including small-cell lung cancer (SCLC) and NSCLC [7]. In NSCLC, the expression of the B7-H3 protein was found in approximately 80% of the studied cases [8], while in SCLC, B7-H3 was detected in nearly 65% of the samples in one study [9] and in 75% of the cases in another study [10]. What is universally accepted is that the expression of the B7-H3 protein in lung cancer tissue is associated with higher metastatic potential [11] and poor clinical outcomes for the patients [7,8,10].

Notably, an immunohistochemical evaluation of B7-H3 expression on lung cancer biopsies derived from patients under diverse antitumor regimens showed a stable expression of B7-H3 in patients with squamous-cell carcinoma (SCC) following treatment [12]. This stable expression of B7-H3 in this particular histological subtype of lung cancer patients makes them perfect candidates for therapies targeting B7-H3. Due to the lower incidence of actionable molecular targets for SCC and the late detection stage, the average five-year survival rate for these patients is low [13]. Additionally, the aforementioned immunohistochemical analysis demonstrated a higher B7-H3 expression in wild-type *epidermal growth factor receptor* (*EGFR*) compared to *EGFR*-mutated adenocarcinoma patients [12]. Therefore, B7-H3 could become a valuable target in these subtypes of lung cancer patients. Interestingly, B7-H3 expression was detected in solid tumors with negligible or negative PD-L1 expression, like pulmonary invasive mucinous adenocarcinoma [14], which highlights that there is room for novel immunotherapeutic options where current ICIs fail.

To be able to take full advantage of B7-H3 protein as a target for lung cancer treatment, we must first elucidate its biological and molecular profile. Although its receptor has not yet been fully unraveled, the multifaceted roles of B7-H3 in tumorigenesis are well established [5]. T-lymphocytes express a receptor unknown to date that binds B7-H3 in antigen-presenting cells (APCs) or tumor cells. This connection supports an immunosuppressive TME, which is molded through the production of associated immunosuppressive cytokines and the polarization of M1 tumor-associated macrophages (TAMs) to an M2 phenotype [5]. On the other hand, B7-H3 possesses pro-tumorigenic capabilities such as tumor migration, invasion, metastasis, treatment resistance, and metabolic reprogramming. B7-H3 is engaged in the potentiation of signal transduction cascades such as phosphatidylinositol-3 kinases (PI3Ks), extracellular signal-regulated kinases (ERKs), and signal transducer and activator of transcription 3 (STAT3) pathways in cancer cells, which may result in augmented tumor cell proliferation and growth [5]. Research efforts are currently trying to decipher these signaling pathways in lung cancer, demonstrating that in NSCLC, B7-H3 triggers epigenetic modifications via the PI3K/AKT pathway, which in turn promotes metastasis through the process of epithelial-to-mesenchymal transition (EMT) [15].

Several B7-H3-based cancer immunotherapy strategies are currently being evaluated in clinical trials enrolling lung cancer patients. Antibody–drug conjugates (ADCs), monoclonal antibodies (mAbs), bispecific antibodies, and chimeric antigen receptor (CAR)-T cells are listed amongst strategic approaches targeting the B7-H3 protein. What is worth mentioning is that the dual checkpoint targeting of B7-H3 and other immune checkpoint molecules is under development [16]. The expression of B7-H3 tends to be mutually exclusive to PD-L1 and cytotoxic T-lymphocyte-associated protein 4 (CTLA4, also known as CD152) expressions [17]. Dual targeting approaches could result in the co-efficient collaboration of the immune system to accelerate tumor shrinkage in solid tumors [16]. Results from a phase I/II clinical trial are encouraging, with an objective response rate (ORR) of 35.7%, progression-free survival (PFS) of 4.83 months, and overall survival (OS) of 12.32 months in NSCLC patients who have not received ICIs in previous therapeutic modalities [16]. However, attention should be drawn, as the dual checkpoint targeting of B7-family proteins is associated with a higher incidence of adverse events, especially immune-related ones [16].

ADCs are shaping the future of cancer treatment. HS-20093, an ADC composed of a human immunoglobulin G1 mAb directed against B7-H3, connected to an as-yet-undefined cytotoxic agent, is currently assessed as monotherapy in a phase I study (NCT05276609) in patients with different solid malignancies, including lung tumors. This study, which recruited 29 patients with NSCLC and 11 patients with SCLC, revealed a favorable toxicity profile accompanied by encouraging anticancer potency, mainly in the SCLC group, where partial responses were attained in 7 out of 9 patients (response rate: 77.8%) [18]. In SCLC, Ifinatamab deruxtecan (I-DXd), a B7-H3-directed ADC comprising an anti-B7-H3 mAb linked to a topoisomerase I inhibitor payload, is under investigation in the phase III IDeate-Lung02 (NCT06203210) clinical trial [19]. Other innovative approaches aiming to exploit B7-H3 protein expression in NSCLC are B7-H3-targeting gold nanocage pH-sensitive conjugates that can precisely attack tumor cells [20], and CAR-engineered natural killer (NK) cells targeting B7-H3 that present enhanced cytotoxicity alongside fewer adverse events [21].

In conclusion, B7-H3 appears to be a promising target that displays elevated and more vigorous expression in lung cancer tissues in comparison to presently available checkpoint protein species. Developing strategies for the synchronous targeting of different proteins of the B7 family could help overcome resistance to current ICIs. Identifying the receptors and intracellular binding domains of the B7-H3 protein in both immune and cancer cells could accelerate this process. Artificial intelligence (AI) is expected to be a valuable ally in this effort, facilitated by AI-driven protein interaction prediction tools [22]. Nevertheless, even though therapeutic modalities targeting B7-H3 in lung cancer are eventually developed, there is an urgent need to identify which subset of patients will most likely profit from them. Shared guidelines assessing B7-H3 expression and quantitative immunostaining scoring systems in different solid tumors are of utmost importance [12]. Delving deeper into the prognostic and predictive role of B7-H3 in lung cancer will provide a patient-tailored and biomarker-guided treatment strategy.
